# Tumor Cell “Slimming” Regulates Tumor Progression through PLCL1/UCP1‐Mediated Lipid Browning

**DOI:** 10.1002/advs.202202011

**Published:** 2022-05-16

**Authors:** Zhiyong Xiong, Wen Xiao, Lin Bao, Wei Xiong, Haibing Xiao, Yan Qu, Changfei Yuan, Hailong Ruan, Qi Cao, Keshan Wang, Zhengshuai Song, Cheng Wang, Wenjun Hu, Zeyuan Ru, Junwei Tong, Gong Cheng, Tianbo Xu, Xiangui Meng, Jian Shi, Zhixian Chen, Hongmei Yang, Ke Chen, Xiaoping Zhang

*Adv. Sci*. **2019**, *6*, 1801862

DOI: 10.1002/advs.201801862

**Figure 5C:**
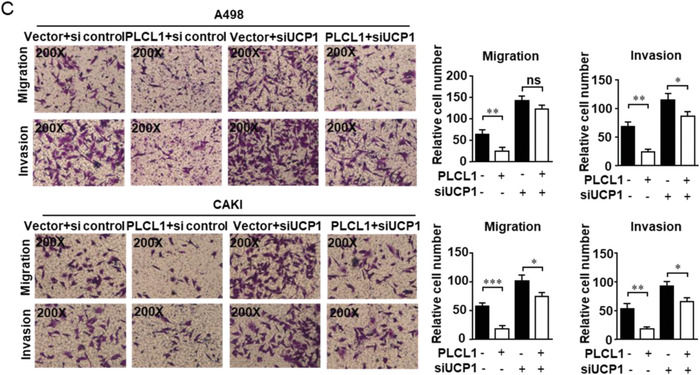
C) Migration and invasion assay for indicated ccRCC cells (Magnification: 200 ×). *t*‐test, ***, *p* < 0.001, **, *p* < 0.01, *, *p* < 0.05, *p* = ns (no significance).

In the original published article, the wrong figure is used for the PLCL1+siUCP1 group in the migration experiment in the CAKI cell experiment in Figure 5C. Please find the correct Figure 5c below. The omissions do not affect the conclusions of the article. The authors apologize for any inconvenience this may have caused.

